# Cellular senescence in skin inflammation: molecular mechanisms and potential clinical implications

**DOI:** 10.3389/fragi.2026.1986107

**Published:** 2026-10-30

**Authors:** Xinhe Wang, Beibei Xiong, Shuxiao Li, Yi Wang, Fulun Li

**Affiliations:** 1 Department of Dermatology, Yueyang Hospital of Integrated Traditional Chinese and Western Medicine, Shanghai University of Traditional Chinese Medicine, Shanghai, China; 2 Department of Dermatology, Shuguang Hospital Affiliated to Shanghai University of Traditional Chinese Medicine, Shanghai, China

**Keywords:** cellular senescence, senescence-associated secretory phenotype (SASP), senolytics, senomorphics, skin inflammation

## Abstract

Cellular senescence is increasingly regarded as a mechanism linking chronic tissue stress to persistent skin inflammation. Senescent cells are defined by stable cell-cycle arrest with remodeling of morphology, metabolism, and secretory phenotype, of which the senescence-associated secretory phenotype (SASP) is central. Because no single marker is sufficient, senescence in skin requires multiparameter, cell-type-resolved criteria. This Review addresses stress sensing, SASP induction, paracrine propagation, immune evasion, cellular heterogeneity, and therapeutic targeting, stratifying evidence by disease: chronic and diabetic wounds are best supported, whereas in psoriasis, atopic dermatitis, cutaneous lupus erythematosus, systemic sclerosis, and vitiligo the evidence remains largely associative, as SASP-like mediators overlap with cytokine programs from activated keratinocytes, stromal cells, and immune cells. Senescence-targeted interventions are classified as senolytics, senomorphics, or senopreventive/geroprotective strategies, and assessed at marker, SASP, senescent-cell-burden, and disease-outcome levels. It further notes that clinical translation depends on defining senescence-maintained lesion subtypes, establishing combinatorial biomarkers and spatial maps, and validating the efficacy and safety window of local therapies in suitable models and human tissues.

## 1 Introduction

The skin is continuously exposed to physical, chemical, and biological stressors, including ultraviolet (UV) radiation, oxidative stress and microbial exposure, making it susceptible to inflammatory disease (Proksch et al., 2008). Inflammatory skin diseases, including chronic cutaneous ulcers, atopic dermatitis (AD), and psoriasis, affect large populations worldwide, and the burden of skin and subcutaneous diseases has increased markedly since 1990 (Li et al., 2026; Mercurio et al., 2020; Skayem et al., 2025). These disorders can disturb sleep, cause pruritus and pain, and are associated with cardiovascular, metabolic, and psychological comorbidities (Pezzolo and Naldi, 2020). Their chronicity, recurrence, and incomplete therapeutic responses suggest persistent tissue-level alterations beyond acute cytokine activation.

Among the biological processes that may contribute to chronic inflammation, cellular senescence has attracted increasing attention. Cellular senescence was first described by Hayflick and Moorhead in 1961 (Hayflick and Moorhead, 1961). It is now understood not as passive cellular aging, but as a stable state of cell-cycle arrest induced by diverse endogenous and exogenous stressors and accompanied by characteristic morphological, metabolic, and secretory changes (Gorgoulis et al., 2019; Hernandez-Segura et al., 2018; López-Otín et al., 2023). A feature of senescent cells is the senescence-associated secretory phenotype (SASP), through which senescent cells release proinflammatory cytokines, chemokines, matrix metalloproteinases, and extracellular vesicles into the surrounding microenvironment (Herranz and Gil, 2018). SASP can act locally or systemically to recruit immune cells, remodel the extracellular matrix (ECM), and induce senescence in neighboring cells. Inflammaging denotes chronic, low-grade, systemic inflammation associated with aging (Franceschi et al., 2000). Cellular senescence and its associated SASP can contribute to this multifactorial state, alongside other processes such as mitochondrial dysfunction and immune dysregulation. Immunosenescence refers to age-related remodeling and functional dysregulation of innate and adaptive immunity, rather than simply cellular senescence in immune cells (Nikolich-Žugich, 2018). Age-related impairment of immune surveillance may allow senescent cells to persist and sustain SASP-mediated inflammation (Majewska and Krizhanovsky, 2025).

Distinguishing senescence as a driver from senescence as a bystander has direct therapeutic implications. Intervention studies in chronic wounds provide evidence that senescent cells can alter disease behavior. In psoriasis, AD, and other inflammatory skin diseases, however, senescence markers and SASP-like mediators are often detectable, but their cellular source and causal position remain uncertain. This Review discusses stress sensing, SASP development, immune evasion, tissue heterogeneity, and therapeutic targeting along the evidence chain connecting cellular senescence with skin inflammation. Our aim is to provide an evidence framework for identifying cutaneous lesion subtypes that may be maintained by senescent cells and for defining potential therapeutic windows.

## 2 Molecular features of cellular senescence in skin

As the body’s first barrier, the skin contains multiple resident cell populations that are continuously exposed to environmental and metabolic stress. Cutaneous cellular senescence is shaped by both endogenous and exogenous factors. Endogenous factors include oxidative stress, telomere shortening, DNA damage, proteostasis disruption, and carbonyl stress, whereas exogenous factors include ultraviolet radiation, particulate pollution, and ionizing radiation (Figure 1).

**FIGURE 1:**
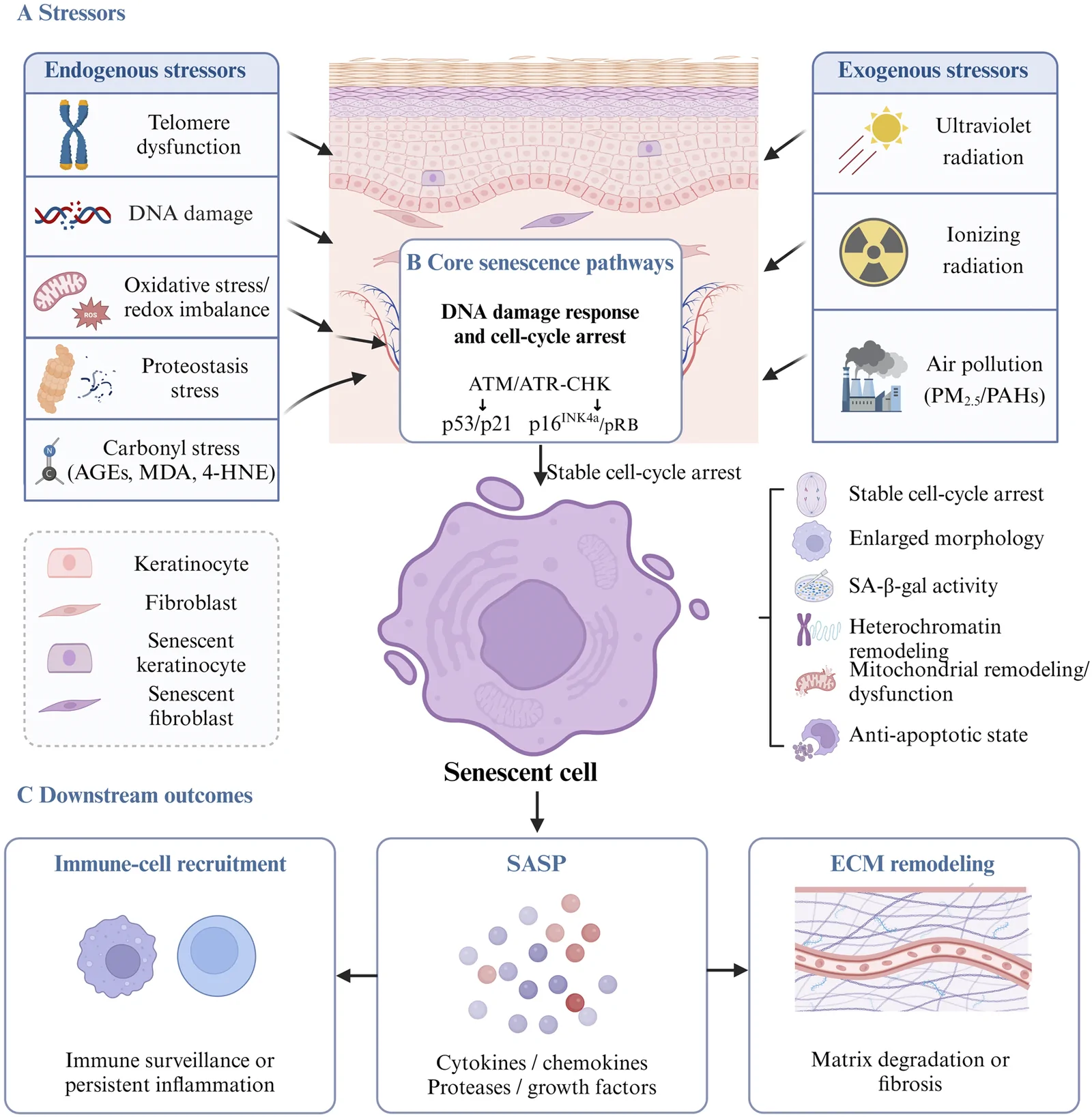
Stressors, core pathways, and downstream outcomes of cellular senescence in skin. **(A)** Endogenous and exogenous stressors engage. **(B)** DNA damage responses and p53/p21 and p16/pRB signaling, promoting stable cell-cycle arrest and senescence-associated phenotypes. **(C)** The senescence-associated secretory phenotype (SASP) contributes to immune-cell recruitment and extracellular matrix (ECM) remodeling. Abbreviations: 4-HNE, 4-hydroxynonenal; AGEs, advanced glycation end products; ATM, ataxia-telangiectasia mutated; ATR, ATM and Rad3-related; CHK, checkpoint kinase; ECM, extracellular matrix; MDA, malondialdehyde; PAHs, polycyclic aromatic hydrocarbons; PM_2.__5_, particulate matter with an aerodynamic diameter ≤2.5 μm; pRB, retinoblastoma protein; ROS, reactive oxygen species; SA-β-gal, senescence-associated β-galactosidase; SASP, senescence-associated secretory phenotype. Created with BioRender.com.

### 2.1 Markers and core regulators of cellular senescence

Cellular senescence can follow replicative exhaustion, oncogene activation, persistent DNA damage, or oxidative stress. The p16^INK4a^/pRB and p53/p21^CIP1^ pathways often regulate stable proliferative arrest, although their expression varies across senescent states (Ahmad et al., 2024; Di Micco et al., 2021). Two interconnected tumor-suppressive pathways, p16^INK4a^/pRB and p53/p21^CIP1^, serve as central molecular hubs that regulate this arrest program (Prieur et al., 2011). Common accompanying features include enlarged morphology, lysosomal expansion, SA-β-gal activity, altered chromatin organization, and apoptosis resistance (Hernandez-Segura et al., 2018).

A single positive marker is insufficient. p16^INK4a^ may be expressed in non-senescent or differentiated cells (Hall et al., 2017), and p21^CIP1^ can indicate a reversible checkpoint response (Barr et al., 2017). SA-β-gal reflects increased lysosomal activity and can also occur in quiescent cells or activated macrophages (Yang and Hu, 2005). Low Ki-67 alone cannot distinguish senescence from quiescence or terminal differentiation (Alessio et al., 2021). Therefore, the panel and relevant negative controls should be reported explicitly, rather than imposing a universal numerical marker threshold.

Senescent cells remain transcriptionally active and can initiate a secretory program centered on SASP, fundamentally altering their relationship with surrounding tissues. Through SASP, senescent cells communicate with immune, stromal, and parenchymal cells. This program can recruit phagocytes for senescent-cell clearance and initiate tissue-repair signals, but when senescent cells persist and accumulate, it can also sustain chronic inflammation (Li et al., 2020; Yin et al., 2022; Zubova et al., 2026).

### 2.2 Endogenous triggers in skin

Endogenous triggers of cutaneous cellular senescence do not act in isolation. Instead, they interact through complex networks that converge on key nodes such as the DNA damage response, cell-cycle arrest, and SASP formation. Telomere dysfunction, DNA damage, redox imbalance, proteostasis disruption, and carbonyl stress act together to shape the senescent phenotype.

Telomere dysfunction is a classical initiating event in replicative senescence. With repeated cell division, telomeres progressively shorten. When telomere-protective structures can no longer maintain chromosome-end homeostasis, telomere ends are recognized as DNA damage sites, thereby activating p53/p21^CIP1^-mediated arrest (Di Micco et al., 2021; Herbig et al., 2004). Because epidermal keratinocytes and dermal fibroblasts experience long-term replicative, mechanical, and inflammatory stress, maintenance of telomere homeostasis is particularly important. Although telomerase activity can partially compensate for telomere erosion, this compensation declines with repeated cell division, and chronic attrition of telomere-protective complexes eventually induces cellular senescence. Recent work showing that Ten1 deficiency in mice induces telomere shortening and models dyskeratosis congenita-related phenotypes further supports a causal link between telomere shortening and cellular senescence (Sanz-Moreno et al., 2025). Thus, telomere dysfunction is an upstream driver of cutaneous cellular senescence rather than a disease-specific cause of any single inflammatory dermatosis.

DNA damage can follow telomere dysfunction, replication stress, or oxidative injury. The canonical double-strand-break response involves recognition by the MRE11-RAD50-NBS1 complex, ATM activation, CHK2 signaling, and stabilization of p53, which can induce p21^CIP1^-mediated checkpoint arrest. In contrast, ATR-CHK1 signaling responds principally to replication stress and replication protein A-coated single-stranded DNA, although the two branches interact (Fan et al., 2025; Gorgoulis et al., 2019). Initial checkpoint arrest can be reversible. Damage repair, apoptosis, or stable senescence depends on the duration and extent of damage and on additional cell-state mechanisms; ATM-p53 activation alone does not establish irreversible arrest. Persistent damage can also promote cytoplasmic chromatin accumulation and cGAS-STING-dependent inflammatory signaling, linking damage sensing to SASP regulation (Di Micco et al., 2021; Gulen et al., 2023).

Redox imbalance is a key endogenous trigger of cellular senescence. Mitochondria continuously generate reactive oxygen species (ROS) during oxidative phosphorylation. Under physiological conditions, these ROS are efficiently removed by antioxidant systems that include SOD2/MnSOD, glutathione peroxidases, and peroxiredoxins. Under pathological conditions, excessive ROS promote p16^INK4a^ upregulation (Wlaschek et al., 2025), activate the NLRP3 inflammasome to release IL-1beta and IL-18 (Latz and Duewell, 2018), and generate reactive lipid peroxidation products such as malondialdehyde (MDA) and 4-hydroxynonenal (4-HNE) (Ayala et al., 2014), ultimately damaging nucleic acids, proteins, and lipids. Studies further show that loss of SOD2 expression and disruption of antioxidant defenses in skin lead to marked senescent-cell accumulation and increased SASP production (Gu et al., 2020; Velarde et al., 2012).

Proteostasis disruption links oxidative injury to tissue functional decline. Skin cells rely on molecular chaperones, the ubiquitin-proteasome system, and autophagy-lysosome pathways to maintain correct protein folding and remove misfolded proteins and damaged organelles. With aging, declining clearance capacity increases the risk of protein misfolding and aggregation (Benoit et al., 2023). Recent studies also indicate that autophagy insufficiency, oxidative stress, and SASP release are dynamically intertwined (Gu et al., 2020). Proteostasis imbalance is both a downstream consequence of ROS and carbonyl stress and a reinforcing mechanism that consolidates cellular senescence through mitochondrial dysfunction, inflammasome activation, and persistent SASP release.

Carbonyl stress links metabolic disturbance to matrix damage and inflammatory signaling. Advanced glycation end products (AGEs) arise through nonenzymatic reactions of reducing sugars or reactive dicarbonyls, including methylglyoxal and glyoxal, with biomolecules (Gugliucci, 2017; Rabbani et al., 2016; Verzijl et al., 2000). Initial glycation products undergo rearrangement, oxidation, and further reactions to generate heterogeneous AGE adducts and cross-links. Lipid peroxidation also generates reactive carbonyls, but many of their products are advanced lipoxidation end products and should not all be classified as AGEs. AGEs can alter ECM properties and activate RAGE-dependent oxidative and inflammatory signaling (Lohwasser et al., 2006; Yu P. et al., 2025). Increased skin and blood AGE measurements in severe psoriasis are associative findings (Papagrigoraki et al., 2017a). Fructose-exposed dermal fibroblasts and GO-AGE/UVB-exposed keratinocytes show senescence-associated or inflammatory readouts, but do not establish that AGE-induced senescent cells maintain human psoriatic lesions (Rella et al., 2025; Sultana et al., 2024). Together, these data support carbonyl stress as an upstream contributor to inflammatory and senescence-like stress responses, although disease specificity and cellular sources require confirmation by spatially and cell-type-resolved studies.

### 2.3 Exogenous triggers in skin

Exogenous triggers arise primarily from the environmental exposures encountered by the skin. They do not constitute independent senescence pathways, but act through endogenous nodes that influence cellular senescence.

Ultraviolet radiation can induce senescence-relevant DNA damage, oxidative stress, MMP activation, and ECM degradation in skin (Fisher et al., 1997). Nuclear and mitochondrial DNA leakage can also activate cGAS-STING and NF-kappaB signaling (Salminen et al., 2025). These observations establish a stress-senescence pathway, but do not by themselves demonstrate that UV-induced senescent cells drive inflammatory dermatoses.

The apparent paradox between the therapeutic benefits of phototherapy and the harmful effects of UV exposure can be understood in terms of wavelength, dose, treatment schedule, and cellular context. Narrowband UVB (NB-UVB) can induce apoptosis of T cells in psoriatic lesions (Ozawa et al., 1999). A randomized controlled trial also showed that NB-UVB improved disease activity in adults with moderate-to-severe atopic eczema compared with a visible-light control (Reynolds et al., 2001). Clinical improvement alone does not demonstrate senolysis or senomorphic activity; such claims require multiparameter identification of senescent target cells and evidence of selective clearance or SASP modulation (Ogrodnik et al., 2024).

Ionizing radiation represents a more direct DNA-damaging exogenous stress. Radiotherapy or other forms of ionizing radiation can cause double-strand breaks, persistent DNA damage responses, and cell-cycle arrest in skin cells, followed by inflammatory cascades. Radiation induces H2A.J-marked senescence and initiates inflammatory responses associated with radiation dermatitis (Abd Al-Razaq et al., 2023; Hippchen et al., 2022). But therapeutic irradiation induces pronounced transcriptional remodeling in skin, with upregulation of inflammatory and immune-regulatory genes and downregulation of genes related to collagen, ECM composition, keratinization, and cornified-envelope formation (Tewary et al., 2024).

Air pollution can amplify cutaneous oxidative stress and barrier injury. PM2.5 can induce mitochondrial injury, cell-cycle arrest, and senescence-like readouts in keratinocytes (Herath et al., 2022; Molagoda et al., 2021; Ryu et al., 2019). Epidemiological studies associate pollution-related exposures with AD and psoriasis (Chen H. L. et al., 2025; Leng et al., 2025), but do not establish lesion-resident senescent cells as the causal source of inflammation. Pollution is therefore best regarded as an upstream modifier rather than disease-specific proof.

## 3 SASP: a mediator linking the senescent state to cutaneous inflammatory signaling

Endogenous and exogenous stressors can be classified as senescent-cell-derived inflammatory secretion only when they induce stable cell-cycle arrest together with a sustained secretory program. SASP is a cell-nonautonomous effect of senescent cells and includes cytokines, chemokines, proteases, growth factors, lipid mediators, extracellular vesicles, and other components (Coppé et al., 2008; Herranz and Gil, 2018). In skin, the boundary between SASP and general inflammation is particularly difficult to define because keratinocytes, fibroblasts, endothelial cells, and immune cells can produce similar mediators during acute or chronic inflammation. SASP should therefore be discussed with reference to cellular state, cellular source, and functional outcome, rather than inferred solely from increased proinflammatory factors.

### 3.1 Multiparameter criteria for defining SASP

An increase in a single secreted factor is insufficient to define a senescence program. Proteomic studies show that SASP comprises numerous soluble proteins and extracellular-vesicle-associated proteins, and that its composition varies by cell type, senescence-inducing stimulus, and time after senescence onset (Basisty et al., 2020). IL-6, IL-1 family cytokines, TNF-alpha, CXCL chemokines, and MMPs are all found in SASP, but acutely activated keratinocytes, dendritic cells, macrophages, and T cells can produce the same mediators. AGE-RAGE signaling illustrates this molecular overlap: it can induce ROS, NF-kappaB activation, IL-1beta, IL-6, TNF-alpha, and MMPs in skin cells, thereby generating a SASP-like inflammatory environment, but activation of this pathway alone does not demonstrate a stable senescent state (Lohwasser et al., 2006; Sultana et al., 2024). In skin tissue, attribution of SASP should rely on combined evidence of cell-cycle arrest or senescence markers, a secretory program, and cell-type or spatial localization (Gorgoulis et al., 2019; Suryadevara et al., 2024).

In inflammatory skin diseases, when only IL-6, TNF-alpha, or MMP upregulation is observed, the more precise description is secretion of inflammatory mediators that overlap with SASP. Only when these mediators occur together with p16^INK4a^, p21^CIP1^, SA-β-gal, senescence transcriptomic signatures, or cell-type -resolved senescent states is it appropriate to further support the term senescent-cell-derived SASP. This distinction reduces the risk of overattributing conventional immune activation to cellular senescence.

### 3.2 SASP induction and maintenance

SASP induction reflects persistent damage sensing rather than transient inflammatory stimulation. Persistent DNA damage responses promote the formation of cytoplasmic chromatin fragments, thereby activating the cGAS-STING axis and driving NF-kappaB- and IRF3-mediated inflammatory transcriptional programs (Dou et al., 2017; Hopfner and Hornung, 2020). Mitochondria can also release mitochondrial DNA through minority mitochondrial outer membrane permeabilization (miMOMP), driving SASP without full apoptosis (Victorelli et al., 2023). DNA damage, redox imbalance, and mitochondrial stress can therefore drive cytokine and chemokine secretion through nucleic-acid-sensing pathways.

SASP composition is not fixed. It is regulated by chromatin state, stress signaling, and feedback circuits. HIRA, PML, and p62/SQSTM1 cooperate to coordinate cytoplasmic-chromatin-associated inflammatory programs (Dasgupta et al., 2024). In oncogene-induced senescence, elevated NOTCH1 activity favors an early TGF-β-rich secretome and restrains the inflammatory program; the later proinflammatory SASP emerges as NOTCH1 activity declines (Hoare et al., 2016). mTOR, JAK-STAT, and p38 MAPK/C/EBPβ regulate secretory output through partly overlapping mechanisms (Han et al., 2022).

SASP factors can help maintain senescence in the producing cells through autocrine feedback and induce senescence in neighboring cells through paracrine signaling. For example, CXCR2-binding chemokines participate in a self-amplifying network that reinforces growth arrest in senescence models (Acosta et al., 2008). SASP components, including TGF-β family ligands, can also induce paracrine senescence in recipient cells (Acosta et al., 2013). These extracellular activities should be distinguished from the intracrine IL-6 mechanism reported by Herbstein (Herbstein et al., 2024). In somatotrophic pituitary tumor cells and therapy-induced senescence models, intracellular IL-6 helped sustain cell-autonomous senescence through a mechanism involving intracellular IL-6 receptors and cGAS–STING–NF-κB signaling, independently of IL-6 secretion and binding to cell-surface IL-6 receptors. The relevance of this intracrine pathway to cutaneous senescence remains to be established.

### 3.3 Tissue effects of SASP in skin

The effects of SASP in skin are time dependent. Short-term, localized SASP can participate in tissue repair: senescent-cell-derived PDGF-AA is required for normal wound healing, and transient SASP exposure can enhance keratinocyte proliferative capacity (Ritschka et al., 2017). Senescence and SASP are therefore not inherently pathological. When senescent cells persist or fail to be cleared, the same secretory program can shift from a repair signal toward chronic proteolysis, inflammatory amplification, and paracrine senescence (Acosta et al., 2013; Saito et al., 2024).

At the tissue level, SASP acts on the major resident cell populations in skin. Keratinocytes may show altered differentiation and barrier-related gene expression, dermal fibroblasts may shift between profibrotic and matrix-degrading programs, and endothelial and resident immune cells participate in angiogenesis, leukocyte recruitment, and inflammatory amplification (Lee et al., 2021). Matrix metalloproteinases such as MMP1 can directly remodel ECM and amplify local inflammation by inducing macrophage TNF-alpha production (Quan et al., 2023). Extracellular vesicles further extend the range of SASP activity; vesicles derived from senescent cells can influence innate immune cell function and transmit SASP signals to neighboring or more distant cell populations (Chen Y. Y. et al., 2024). Thus, the tissue effects of SASP are not determined by a single secreted factor, but by the source of senescent cells, the response properties of target cells, the clearance capacity of the microenvironment, and the dynamic interactions among these elements.

### 3.4 Evidence stratification across skin diseases

Evidence linking cellular senescence to inflammatory skin disease remains uneven. Lesional p16^INK4a^, p21^CIP1^, SA-β-gal activity, or senescence-associated transcriptional signatures provide associative evidence, but do not establish stable arrest or pathogenicity. Stronger inference requires cell-type localization, multiparameter validation, disease-relevant SASP or functional effects, and improvement after selective perturbation. Few diseases currently satisfy this complete chain.

Chronic and diabetic wounds provide the most integrated evidence. Human wounds show increased p16/p21 and senescence/SASP programs, with higher CDKN1A associated with delayed healing (Samarawickrama et al., 2024; Yu et al., 2024). Experimental studies implicate CXCR2-mediated macrophage-fibroblast signaling and persistent senescent fibroblasts, while ABT-263 or DPP4-targeted clearance improves repair (Samarawickrama et al., 2024; Wilkinson et al., 2019). However, the dominant pathogenic population in human wounds and the safe therapeutic window remain undefined.

In psoriasis, lesional keratinocytes and CD4^+^ T cells show senescence-associated phenotypes. IGFBP2-p21 and RAS-PI3K/AKT signaling provide keratinocyte-specific mechanisms, whereas topical ABT-737 and CD4-specific Tet2 deletion improve psoriasiform dermatitis in mice (Mercurio et al., 2024; Mercurio et al., 2020; Zhu et al., 2024). These findings provide functional and preclinical interventional evidence, although human senescence-selective trials are absent. Evidence in atopic dermatitis (AD) is less mature and largely restricted to elderly AD. Fibroblast- and SASP-enriched programs are associated with macrophage and NK-cell recruitment, and fibroblast experiments support enhanced immune-cell chemotaxis (Luo et al., 2024).

Cutaneous lupus erythematosus (CLE) has a comparatively coherent evidence chain: human p21-high basal epidermal cells are linked to type I interferon amplification and HLA-I remodeling, while fisetin or topical ABT-737 improves lupus-related skin disease in mice (Jiang et al., 2024; Yamamoto et al., 2025). In systemic sclerosis (SSc), multiparameter and spatial studies support senescent dermal fibroblasts linked to inflammatory and profibrotic outputs, but lineage-selective *in vivo* causality and clinical efficacy remain unproven (Chiu et al., 2023; Kizilay Mancini et al., 2022; Shi et al., 2023). Vitiligo evidence remains preliminary: lesional fibroblasts show multiple senescence-associated features, whereas melanocyte evidence relies mainly on p16^INK4a^ positivity and is partly confounded by anatomical site or sun exposure (Lee et al., 2020; Rani et al., 2017). Therefore, cellular senescence should not be assigned an equivalent causal role across diseases; detailed comparisons of implicated cell populations, validation methods, pathogenic effects, and limitations are provided in Table 1.

**TABLE 1:** Disease-specific evidence linking cellular senescence to skin diseases.

Disease	Senescent cell population and identification evidence	Senescence-linked pathogenic mechanisms	Functional or interventional evidence
Chronic or diabetic wounds	Human chronic wounds and diabetic foot ulcers (DFUs) show increased CDKN1A/p21 and CDKN2A/p16, senescence-gene and SASP signatures, and an association between higher p21 and slower healing. Bulk DFU transcriptomics also identified coordinated upregulation of CDKN1A, CXCL8, IGFBP2, IL1A, MMP10, SERPINE1, and TGFA, although cellular sources could not be resolved. Experimental studies identify dermal fibroblasts and persistent SASP-producing macrophages as candidate effectors (Samarawickrama et al., 2024; Wilkinson et al., 2019; Yu et al., 2024)	A CXCR2-enriched macrophage SASP can induce paracrine p21 in fibroblasts. In diabetic models, reduced PDK4 promotes fibroblast senescence through impaired glycolysis and altered YAP/JNK signaling, whereas reduced NCOA4-dependent ferritinophagy permits senescent fibroblasts to resist ferroptotic removal. These programs are associated with defective re-epithelialization, matrix deposition, and inflammatory resolution (Wei et al., 2023; Wilkinson et al., 2019)	ABT-263 reduced senescence markers and accelerated healing in diabetic mice. DPP4-targeted PARP1 nanospheres preferentially eliminated senescent fibroblasts and improved tissue repair. PDK4 restoration or pharmacological induction of ferroptosis also improved experimental healing (Samarawickrama et al., 2024; Wei et al., 2023)
Psoriasis	Human lesions contain p16^INK4a^/p21-high CD4^+^ T cells identified by multispectral immunohistochemistry. Patient-derived lesional keratinocytes show SA-β-gal activity, p16/p21 and IGFBP2 expression, apoptosis resistance, and RAS-AKT activation associated with p53/p21-mediated senescence (Mercurio et al., 2024; Mercurio et al., 2020; Zhu et al., 2024)	Intracellular IGFBP2 stabilizes cytoplasmic p21 and sustains apoptosis resistance. RAS-PI3K/AKT signaling maintains keratinocyte senescence, while senescence-associated changes in CD4^+^ T cells intersect with the TET2-Th17 pathway (Mercurio et al., 2024; Mercurio et al., 2020; Zhu et al., 2024)	IGFBP2 or p21 knockdown restored keratinocyte apoptosis *in vitro*. AKT inhibition reduced cytokine-induced senescence and inflammation in a RAS-driven psoriasiform model. Topical ABT-737 and CD4-specific Tet2 deletion also reduced disease severity in mice (Mercurio et al., 2024; Mercurio et al., 2020; Zhu et al., 2024)
Atopic dermatitis	Elderly AD lesions show fibroblast- and SASP-enriched transcriptomic modules with fibroblast, macrophage, and NK-cell co-localization. RNA sequencing, immunofluorescence, qRT-PCR, flow cytometry, and chemotaxis assays were used, although stable cell-cycle arrest was not verified in the same lesional cells (Luo et al., 2024)	Senescent or inflammation-exposed fibroblasts increase macrophage and NK-cell chemotaxis. A murine AD model linked disturbed BCAA metabolism to p21-high inflammatory senescence and Th2 activity (Luo et al., 2024; Wang Y. et al., 2025)	Topical Liuweirunfu oil reduced relapse in a randomized clinical study. Reduced p21, restoration of BCAT1-mediated BCAA catabolism, and suppression of Th2 responses were demonstrated in the accompanying murine model rather than as senescent-cell clearance in patients (Wang Y. et al., 2025)
Cutaneous lupus erythematosus	Human CLE lesions contain increased p16+ basal progenitor cells and p21+ basal or suprabasal transient-amplifying cells. Bulk transcriptomics and scRNA-seq further identify CDKN1A-high epidermal basal cells, supported by *in vitro* and mouse validation (Wang et al., 2022; Yamamoto et al., 2025)	Senescent basal cells amplify type I IFN signaling in neighboring epidermis. EGFR-associated HLA-I downregulation may permit immune evasion of senescent cells while increasing HLA-I-dependent cytotoxic injury to neighboring basal cells (Yamamoto et al., 2025)	Fisetin improved skin disease in MRL/lpr mice. Topical ABT-737 reduced senescence-associated cells, inflammation, immune-complex deposition, and lesions in a humanized CLE mouse model (Jiang et al., 2024; Yamamoto et al., 2025)
Systemic sclerosis	SSc skin shows p16+ spindle-shaped dermal cells, vimentin + telomere-associated DNA-damage foci, and senescence-gene enrichment across fibroblast, vascular, pericyte, and myeloid compartments. An independent biopsy study defined senescent fibroblasts as p16- and/or p21-positive and Ki67-negative; their abundance correlated with fibrosis, inflammation, fibroblast CCN2 expression, and EndMT (Chiu et al., 2023; Kizilay Mancini et al., 2022; Shi et al., 2023)	Patient-derived fibroblasts exhibit ROS and mitochondrial dysfunction, DNA-damage responses, IL-6/IL-8 secretion, and profibrotic gene expression. Tissue senescence scores correlate spatially with ECM programs (Chiu et al., 2023; Kizilay Mancini et al., 2022; Shi et al., 2023)	ROS scavengers reduced inflammatory and fibrotic outputs in SSc fibroblasts *in vitro*. Changes in vascular p21, uPAR, and EndMT accompanied clinical improvement after cyclophosphamide or autologous hematopoietic stem-cell transplantation, but neither treatment establishes senescence-selective clearance (Chiu et al., 2023; Kizilay Mancini et al., 2022; Shi et al., 2023)
Vitiligo (autoimmune inflammatory depigmenting disease)	Lesional fibroblasts from patients with non-segmental vitiligo show enlarged morphology, SA-β-gal activity, and increased p16, p21, and HP1. Human perilesional epidermis also contains more p16^INK4a^-positive melanocytes identified by p16^INK4a^/MITF co-localization, although p16^INK4a^ was used as a single senescence marker (Lee et al., 2020; Rani et al., 2017)	IFN-γ induces ROS-JAK2-STAT1-p21-dependent melanocyte senescence with IL-6 and HSP70 release. Under psychological-stress conditions, an IL-6/JAK2-STAT3 autocrine loop reinforces fibroblast senescence and promotes melanocyte apoptosis (Cao et al., 2025; Wang et al., 2014)	p21, JAK2, or STAT1 knockdown and N-acetylcysteine attenuated melanocyte senescence *in vitro*; IL-6R blockade reduced stress-associated fibroblast senescence in experimental models. No senescence-selective *in vivo* or clinical intervention has been established (Cao et al., 2025; Wang et al., 2014)

Abbreviations: ABT-263, navitoclax; AD, atopic dermatitis; AKT, protein kinase B; BCAA, branched-chain amino acid; BCAT1, branched-chain amino-acid transaminase 1; CCN2, cellular communication network factor 2; CLE, cutaneous lupus erythematosus; CXCL, C-X-C motif chemokine ligand; CXCR2, C-X-C motif chemokine receptor 2; DFU, diabetic foot ulcer; DPP4, dipeptidyl peptidase 4; ECM, extracellular matrix; EGFR, epidermal growth factor receptor; EndMT, endothelial-to-mesenchymal transition; HLA-I, human leukocyte antigen class I; HP1, heterochromatin protein 1; IFN, interferon; IGFBP2, insulin-like growth factor-binding protein 2; IL, interleukin; IMQ, imiquimod; JAK, janus kinase; LWRF, liuweirunfu; MITF, microphthalmia-associated transcription factor; NCOA4, nuclear receptor coactivator 4; NK, natural killer; PARP1, poly (ADP-ribose) polymerase 1; PDK4, pyruvate dehydrogenase kinase 4; PI3K, phosphoinositide 3-kinase; qRT-PCR, quantitative reverse-transcription polymerase chain reaction; RAS, rat sarcoma virus protein; ROS, reactive oxygen species; SA-β-gal, senescence-associated β-galactosidase; SASP, senescence-associated secretory phenotype; scRNA-seq, single-cell RNA sequencing; SSc, systemic sclerosis; STAT, signal transducer and activator of transcription; TET2, ten-eleven translocation 2; Th, T helper; uPAR, urokinase plasminogen activator receptor; YAP, Yes-associated protein.

## 4 The senescence inflammation cycle in skin

Persistent SASP can shift senescent cells from a local injury response to a maintaining factor in chronic inflammation. In skin, this process is shaped mainly by three linked mechanisms: paracrine senescence extends injury signals to neighboring cells, insufficient immune clearance allows senescent cells to persist, and compartment-specific cellular heterogeneity determines inflammatory patterns and tissue outcomes (Figure 2).

**FIGURE 2:**
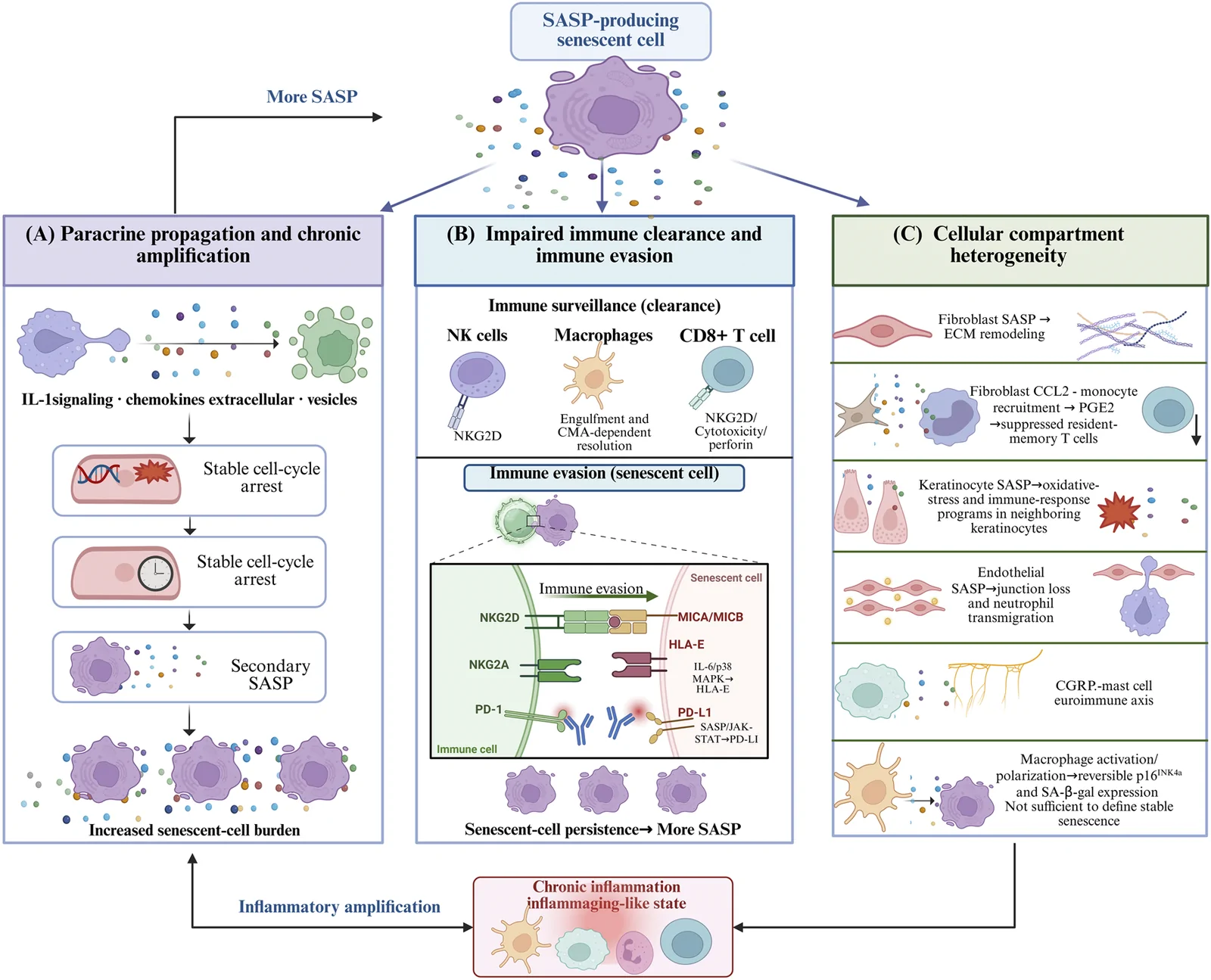
The senescence–inflammation cycle in skin. **(A)** The senescence-associated secretory phenotype (SASP) promotes paracrine senescence and secondary SASP production, increasing senescent-cell burden and inflammatory amplification. **(B)** Natural killer cells, macrophages, and CD8^+^ T cells contribute to senescent-cell clearance. PD-L1–PD-1 signaling, HLA-E–NKG2A-mediated inhibition, and ADAM10-mediated MICA/MICB shedding can impair clearance, permitting senescent-cell persistence. **(C)** Compartment-specific effects include fibroblast-mediated matrix remodeling and CCL2–monocyte–PGE_2_-dependent suppression of resident-memory T cells; keratinocyte oxidative-stress and immune-response programs; and endothelial junction loss, neutrophil transmigration, and associated CGRP–mast cell signaling. Reversible p16^INK4a^ and SA-β-gal expression in activated macrophages does not alone establish stable senescence. Together, these context-dependent processes reinforce SASP production, tissue stress, and chronic inflammation, sustaining an inflammaging-like state. Abbreviations: ADAM10, a disintegrin and metalloproteinase 10; CCL2, C-C motif chemokine ligand 2; CGRP, calcitonin gene-related peptide; CMA, chaperone-mediated autophagy; ECM, extracellular matrix; HLA-E, human leukocyte antigen E; IL, interleukin; JAK, Janus kinase; MAPK, mitogen-activated protein kinase; MICA/MICB, major histocompatibility complex class I chain-related proteins A and B; NK, natural killer; NKG2A, natural killer group 2 member A; NKG2D, natural killer group 2 member D; PD-1, programmed cell death protein 1; PD-L1, programmed death-ligand 1; PGE_2_, prostaglandin E_2_; SA-β-gal, senescence-associated β-galactosidase; SASP, senescence-associated secretory phenotype; STAT, signal transducer and activator of transcription. Created with BioRender.com.

### 4.1 Paracrine propagation and chronic amplification

After senescent cells accumulate, the local microenvironment can shift from transient repair toward low-grade, persistent inflammation. Cytokines, chemokines, and proteases within SASP can recruit and activate myeloid cells, which in turn release inflammatory mediators and matrix-degrading enzymes, creating a tissue context resembling inflammaging (Ferrucci and Fabbri, 2018). Studies of chronic wounds show that persistent inflammation, increased protease activity, and impaired ECM remodeling are important features of stalled healing (Pastar et al., 2023; Pulido et al., 2021). The pathological relevance of SASP therefore lies not in the elevation of any single factor, but in whether it maintains a local injury response as a self-perpetuating tissue state.

Paracrine senescence is a key mechanism by which this state expands. Senescent cells can influence neighboring cells through IL-1-related inflammatory programs, chemokines, and extracellular vesicles, causing non-senescent cells to acquire DNA damage responses, cell-cycle arrest, or SASP secretion (Acosta et al., 2013; Chen Y. Y. et al., 2024). In chronic wounds, increased senescence markers and SASP-related transcripts are associated with delayed healing; in a cohort of 79 patients with chronic wounds, Cox modeling showed that high CDKN1A expression predicted prolonged healing time (Yu et al., 2024). In contrast to the beneficial role of senescent cells in acute wounds, pharmacological clearance of senescent cells improves wound repair in animal models (Samarawickrama et al., 2024). DPP4-targeted nanospheres can selectively eliminate senescent fibroblasts in diabetic wounds, downregulate SASP, induce M2 macrophage polarization, and accelerate re-epithelialization and collagen deposition, supporting the concept that reducing senescent-cell burden can improve the diabetic wound microenvironment (Zhao et al., 2022). By comparison, links between senescence and inflammatory skin diseases such as psoriasis and AD mainly suggest involvement of metabolic-inflammatory pathways, including AGE/RAGE signaling, rather than a proven senescence-driven mechanism (Papagrigoraki et al., 2017a; Wang et al., 2024).

### 4.2 Impaired immune clearance and immune evasion

Natural killer (NK) cells, macrophages, and CD8^+^ T cells all participate in immune surveillance and clearance of senescent cells (Majewska and Krizhanovsky, 2025). In autologous human dermal fibroblast co-cultures, both NK and CD8^+^ T cells targeted senescent fibroblasts through NKG2D-dependent recognition (Pereira et al., 2019). Macrophages contribute through engulfment; macrophage-specific disruption of chaperone-mediated autophagy impaired clearance and delayed senescence resolution during mouse wound healing (Sereda et al., 2026). With aging, immune surveillance can become less effective. In aged mice, CD8^+^ T-cell cytotoxicity was reduced, and mice lacking perforin accumulated more senescent cells and developed chronic inflammation (Ovadya et al., 2018).

Senescent cells can also evade immune recognition and killing. SASP-driven JAK/STAT signaling can upregulate PD-L1 in human fibroblast models. PD-L1-positive senescent cells resist CD8^+^ T-cell surveillance. (Onorati et al., 2022). Some senescent cells may also reduce NKG2D-dependent NK-cell recognition through ADAM10-mediated MICA/MICB shedding (Zhang et al., 2022). In human dermal fibroblasts, HLA-E expression is regulated by p38 MAPK and can be induced by the SASP factor IL-6. HLA-E binds the inhibitory receptor NKG2A on NK cells and highly differentiated CD8^+^ T cells, thereby suppressing immune clearance. HLA-E+ senescent cells are enriched in skin from elderly donors (Pereira et al., 2019). Impaired immune surveillance and active immune evasion may therefore contribute to senescent-cell persistence, but their relative contributions to inflammatory skin diseases remain to be established.

### 4.3 Cellular compartment heterogeneity

Cutaneous senescence is not distributed across heterogeneous cell states rather than carried uniformly by one population. Lineage, anatomic compartment, inducing stress, and time after senescence onset can each reshape SASP composition and immunogenicity (Basisty et al., 2020; Kwiatkowska et al., 2023). Single-cell RNA sequencing has identified at least six human epidermal basal-cell subpopulations with distinct stress-response features, and four major dermal fibroblast subpopulations whose priming state declines with age (Solé-Boldo et al., 2020; Zou et al., 2021). Compartmental differences are also evident across diseases: single-cell analysis indicates that fibroblasts are a major source of proinflammatory and vasodilatory signals in rosacea (Chen M. et al., 2024), whereas in radiation dermatitis models, endothelial cells, fibroblasts, keratinocytes, and monocytes/macrophages can all show senescence-associated changes, with monocytes/macrophages exhibiting the highest senescent fraction (Chen Y. et al., 2025).

SASP secretion from different compartments corresponds to different tissue outcomes. SASP from fibroblast-dominant compartments may influence ECM remodeling and stromal-immune crosstalk (Solé-Boldo et al., 2020). In aged human skin subjected to intradermal challenge, CCL2-expressing senescent fibroblasts recruited inflammatory monocytes whose prostaglandin E2 production suppressed resident memory T-cell activation and proliferation (Chambers et al., 2021). Keratinocyte senescence is accompanied by proinflammatory secretory changes that can affect neighboring cells. UVA-induced senescent primary human keratinocytes released a proinflammatory secretome that elicited oxidative-stress and immune-response programs in HaCaT cells (Valerio et al., 2021). SASP from senescent endothelial cells can weaken intercellular junctions, compromise vascular barrier integrity, and promote neutrophil adhesion and transendothelial migration (Najari Beidokhti et al., 2025). In the skin, a mouse model of endothelial cell senescence further implicated this secretory program in activation of the CGRP–mast cell neuroimmune axis, contributing to dermal thinning, collagen degradation, and delayed wound healing (Wicaksono et al., 2025). Immune-cell states require separate interpretation because macrophage p16^INK4a^ and SA-β-gal can be induced reversibly during physiological polarization rather than indicating stable senescence (Hall et al., 2017). Stable arrest, lineage localization, and functional secretory evidence are therefore needed to distinguish a senescent immune population from conventional activation.

This heterogeneity has direct therapeutic implications. Senolytic sensitivity varies among senescent cell types, as illustrated by the differential activity of dasatinib and quercetin across human cell models (Zhu et al., 2015). A treatment active against one lesion-resident population may therefore spare another or alter only part of the SASP network. Future studies should combine lineage-aware marker panels and spatial mapping with pre- and post-treatment assessment to determine which senescent population is removed or functionally suppressed.

## 5 Senescence-targeted therapeutic strategies in skin disease

In skin diseases, senescence-targeted therapies should be classified into three categories according to their mechanisms of action and required evidence endpoints: senolytics, senomorphics, and senopreventive/geroprotective strategies. Senolytics selectively induce senescent cell death by exploiting their dependence on anti-apoptotic pathways. Senomorphics aim to modify the secretory phenotype of already senescent cells, ideally without directly killing them. Senopreventive/geroprotective strategies aim to prevent the establishment of senescence before or during stress exposure (Figure 3).

**FIGURE 3:**
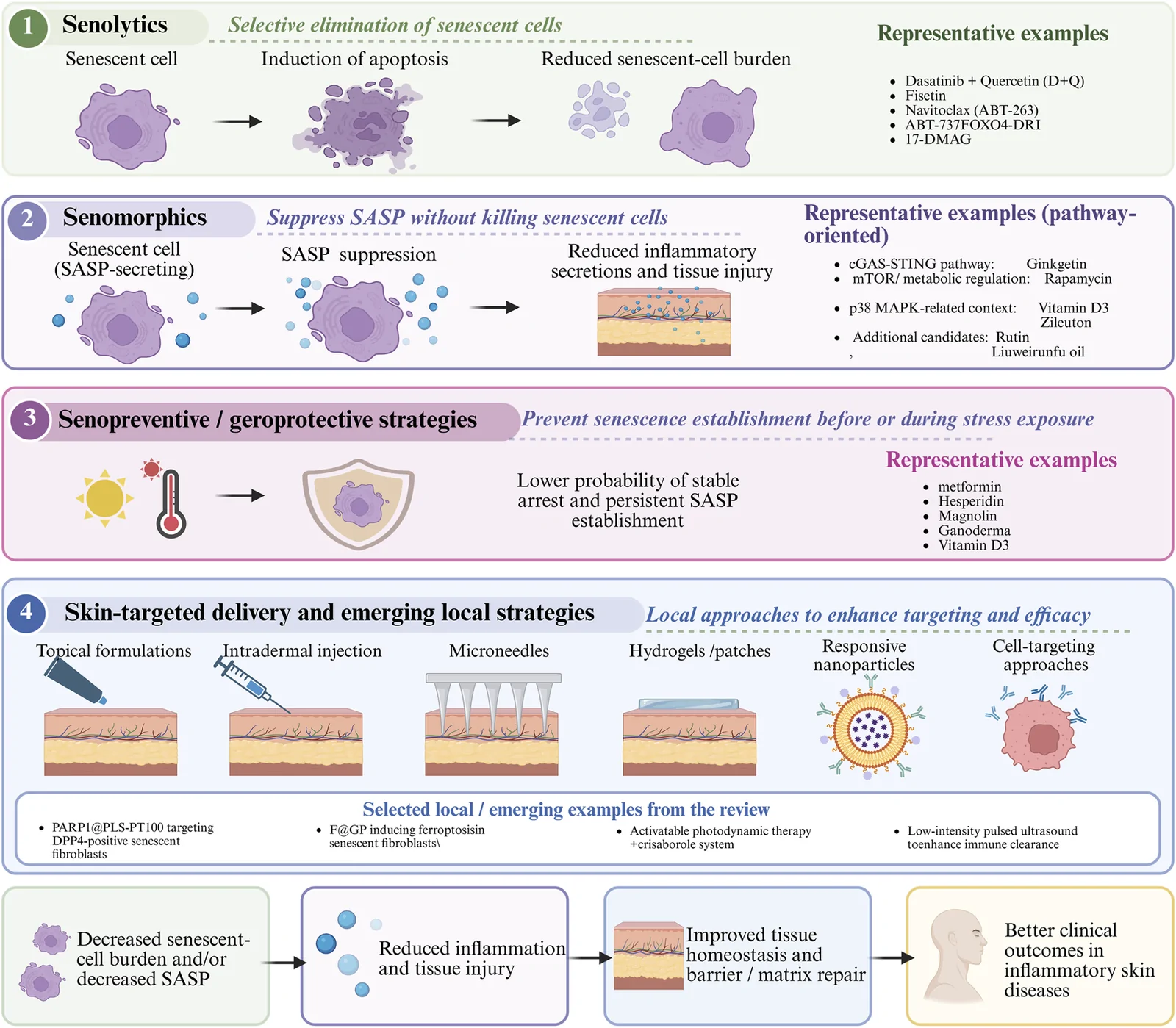
Senolytic, senomorphic, senopreventive/geroprotective, and skin-targeted therapeutic strategies. Senolytics selectively eliminate senescent cells by exploiting their dependence on pro-survival and anti-apoptotic pathways, thereby reducing senescent-cell burden rather than merely suppressing downstream inflammatory mediators. Senomorphics modify the secretory phenotype of already senescent cells, ideally without directly killing them, with the aim of reducing SASP-mediated inflammatory signaling and tissue injury while preserving potentially beneficial transient senescence responses. Senopreventive/geroprotective strategies aim to prevent the establishment of senescence before or during stress exposure, for example by preventing stress-induced p16^INK4a^ or SA-β-gal expression, rather than by clearing already senescent cells or modifying their secretory phenotype. Skin-targeted approaches, including topical formulations, intradermal administration, microneedles, hydrogels or patches, responsive nanoparticles, and cell-targeting systems, may improve local drug exposure and reduce systemic toxicity. Emerging strategies additionally combine lesion-directed senescent-cell targeting with ferroptosis induction, photodynamic or anti-inflammatory treatment, or enhancement of local immune clearance. Collectively, these interventions are intended to reduce senescent-cell burden and/or SASP activity, attenuate inflammation and tissue injury, and restore epidermal barrier and extracellular-matrix homeostasis. Abbreviations: DPP4, dipeptidyl peptidase 4; ECM, extracellular matrix; PDE4, phosphodiesterase 4; SASP, senescence-associated secretory phenotype; SA-β-gal, senescence-associated β-galactosidase. Created with BioRender.com.

According to evidence endpoints, this review classifies intervention strategies at the marker level (reduction in p16^INK4a^, p21^CIP1^, SA-β-gal, *etc.*), the SASP level (reduction in senescence-associated secretory factors such as IL-6 and MMPs), the senescent-cell-burden level (reduction in senescent cell number or proportion shown by single-cell or spatial omics), and the disease-outcome level (improvement in healing, inflammation, relapse, *etc.*). These are summarized in Table 2.

**TABLE 2:** Evidence hierarchy of candidate senolytic, senomorphic, and senopreventive/geroprotective strategies in dermatology.

Class	Agent/strategy	Proposed mechanism	Senescence-specific endpoint	Dermatological evidence level	References
Senolytic	Dasatinib	Tyrosine kinase/PI3K pathway inhibition	Reduced senescent-cell burden and p16-linked fibrosis signals in keloid fibroblasts/tissue	Direct cutaneous: human keloid tissue, cultured keloid fibroblasts, xenograft model	(Darmawan et al., 2023)
Senolytic	D + Q	Disables senescent-cell pro-survival networks through tyrosine kinase, PI3K/AKT, and glucose-transporter pathways	Skin chimera: senescent fibroblast clearance; extra-cutaneous trials: senescence-biomarker changes	Direct cutaneous preclinical plus extra-cutaneous human feasibility	(Farr et al., 2024; Hickson et al., 2020; Takaya and Kishi, 2024)
Senolytic/senomorphic	Fisetin	Flavonoid with senolytic and SASP-modulating activity	Reduced senescent dermal fibroblast burden and SASP-associated signals in skin chimera	Anti-inflammatory efficacy in psoriasis model; senolytic causality not established	(Takaya et al., 2024)
Senolytic	Navitoclax (ABT-263)	BCL-2/BCL-XL inhibition	Selective clearance of senescent dermal fibroblasts; reduced aged-skin senescence; improved diabetic/aged wound healing	Human fibroblasts, human-skin chimera, mouse skin and wound models	(Kim et al., 2022; Samarawickrama et al., 2024; Shvedova et al., 2024)
Senolytic	ABT-737	BH3-mimetic inhibition of BCL-2, BCL-XL and BCL-w	Reduced p16/p21-positive senescent cells in IMQ lesions and UV/photoaging models	Mouse cutaneous disease/photoaging models	(Kim et al., 2022; Zhu et al., 2024)
Senolytic	A1331852/A1155463	Selective BCL-XL inhibition	Senescent-cell selective apoptosis in HUVEC and IMR90 senescence models	*In vitro* senescence models; no direct skin-disease model	(Zhu et al., 2017)
Senolytic	FOXO4-DRI	Disrupts FOXO4-p53 interaction and apoptosis resistance	Apoptosis of senescent fibroblasts; p16/p53-pS15-linked keloid senescence signals	Scar/keloid-related cell and tissue models	(Baar et al., 2017; Kong et al., 2025)
Senolytic	17-DMAG	HSP90 inhibition and AKT survival-pathway destabilization	Senescent-cell killing in multiple induced senescence models; reduced p16^INK4A^ in progeroid mice	Extra-cutaneous progeroid and cell models	(Fuhrmann-Stroissnigg et al., 2017)
Candidate senotherapeutic	Gingerenone A//I3C	BCL-XL/BCL-2 modulation, antioxidant defense, PI3K/AKT/mTOR suppression, or apoptosis induction	Variable senescent-cell apoptosis, p16, SASP, or senescence-associated gene readouts	Mostly *in vitro* or non-skin aging models; limited dermal fibroblast evidence	(Kühnel et al., 2025; Moaddel et al., 2022; Sax et al., 2024; Zhang et al., 2023)
Senomorphic candidate	Ginkgetin	Direct STING binding and cGAS-STING signal suppression	Reduced p16/p21 and SASP cytokines in experimental aging models	Mostly extra-cutaneous/multi-tissue senescence models	(Liu et al., 2025)
Senomorphic	Rapamycin	mTOR inhibition	Human skin: reduced epidermal p16^INK4A^ and increased collagen VII; experimental reduction of SA-β-gal/MMP1	Direct human aged-skin trial plus skin cell/UVB models	(Chung et al., 2019; Qin et al., 2018; Tang et al., 2024)
Candidate senomorphic/Senopreventive/geroprotective strategies	Vitamin D3	p38 MAPK inhibition in senescent fibroblasts	Reduced SASP mediator secretion from senescent fibroblasts	*In vitro* senescent fibroblast model with human-skin immune relevance	(Sayegh et al., 2024)
Candidate senomorphic	Zileuton	5-lipoxygenase inhibition with p38/SASP modulation	Reduced senescence-associated secretory markers and radiation-ulcer injury	Radiation-induced cutaneous ulcer model and cells	(Park et al., 2022)
Candidate senomorphic	Rutin	Broad SASP suppression	Downregulation of representative SASP factors in senescent cells	Cell and tumor-microenvironment senescence models	(Liu et al., 2024)
Candidate senomorphic/metabolic	Liuweirunfu Oil	Restores BCAA metabolism and modulates inflammatory senescence	Lowered p21 and SASP factors (IL-8, IL-11) in AD-related mechanistic analyses	Human AD relapse study plus mechanistic models	(Wang Y. et al., 2025)
Senopreventive/geroprotective strategies	Metformin	Mitophagy and PI3K/AKT/mTOR modulation	Reduced UVA-induced senescence features in fibroblasts	Human foreskin fibroblast and mouse photoaging models	(Chen et al., 2022)
Senopreventive/geroprotective strategies	Hesperidin	Mitochondrial protection and oxidative-stress modulation	Reduced PM2.5-induced cell-cycle arrest and cellular senescence in HaCaT keratinocytes	Human keratinocyte pollution-stress model	(Herath et al., 2022)
Senopreventive/geroprotective strategies	Magnolin	CXCL10/p38 pathway modulation	Reduced β-gal activity, p16/p21, and MMP expression in skin-aging models	Human fibroblast, *ex vivo* human skin, and facial-skin aging evidence	(Wang C. et al., 2025)
Senopreventive/geroprotective strategies	Ganoderma lucidum extract	Antioxidant defense; cytoprotection; p16-related transcriptional modulation	Modulation of p16-related signals; no direct senescent-cell clearance	Preclinical; human dermal fibroblasts	(Kühnel et al., 2025)

### 5.1 Senolytics: selective elimination of senescent cells

The therapeutic potential of senolytics derives from the dependence of senescent cells on anti-apoptotic networks. Senolytic activity should be defined by reduced senescent-cell burden, not merely by downstream anti-inflammatory effects. BCL-2 family signaling, PI3K/AKT, FOXO4-p53 interactions, and HSP90-dependent kinase stabilization can all maintain senescent-cell survival. Blocking these pathways may eliminate pathological cell populations that persist in skin tissue, thereby reducing SASP-mediated inflammatory amplification, matrix degradation, and stalled repair.

Dasatinib plus quercetin (D + Q) remains the best-known senolytic combination. Dasatinib inhibits multiple tyrosine kinases, including ephrin and platelet-derived growth factor receptors, whereas quercetin affects PI3K/AKT and glucose-transporter pathways. In a human-mouse skin chimera model, D + Q selectively cleared senescent fibroblasts without showing comparable toxicity toward non-senescent cells (Takaya and Kishi, 2024). Dasatinib monotherapy also reduced senescent-cell burden and fibrosis in human keloid tissue (Darmawan et al., 2023), suggesting potential value in lesions dominated by matrix remodeling. However, extracutaneous D + Q trials have established the feasibility of intermittent dosing and biomarker modulation, but have not demonstrated efficacy in inflammatory skin disease. Therefore, D + Q should be classified as a senolytic candidate with skin-aging and keloid evidence, but not yet as an established therapy for inflammatory skin diseases.

Fisetin is another natural flavonoid with translational potential. In a human-mouse skin chimera system, fisetin reduced the burden of senescent dermal fibroblasts and improved features of dermal aging (Takaya et al., 2024). In an imiquimod (IMQ)-induced psoriasis-like inflammation model, fisetin also attenuated epidermal thickening, aberrant keratinocyte proliferation, and Th17-type inflammation through mTOR/IL-17A- and autophagy-related pathways (Roy et al., 2022). Thus, fisetin has senolytic evidence in skin aging models and anti-inflammatory activity in a psoriasis-like inflammation model. Whether the latter therapeutic benefit results directly from selective senescent-cell clearance remains unproven.

BCL-2/BCL-XL inhibitors represent another important senolytic class. Many senescent cells persist despite ongoing damage and pro-apoptotic stress because anti-apoptotic proteins such as BCL-2 and BCL-XL maintain their survival. Releasing this survival dependence can therefore push senescent cells into apoptosis. Studies of mitochondrial stress further suggest that some senescent cells can release mitochondrial DNA and drive SASP without completing apoptosis, linking anti-apoptotic networks to SASP maintenance (Victorelli et al., 2023). Navitoclax (ABT-263) and ABT-737 induce apoptosis of senescent fibroblasts *in vitro* and clear UV-induced senescent dermal fibroblasts in mouse skin (Kim et al., 2022; Shvedova et al., 2024). Topical ABT-737 also reduces senescent-cell burden and improves IMQ-induced psoriasiform dermatitis in mice (Zhu et al., 2024). In a type 2 diabetic mouse model, pharmacological senolytic clearance similarly improves wound-healing kinetics and tissue architecture (Samarawickrama et al., 2024). The selective BCL-XL inhibitors A1331852 and A1155463, designed on the basis of BCL-XL crystal structure, provide more restricted target engagement, although their evidence is largely derived from *in vitro* senescence models rather than skin-disease models (Zhu et al., 2017).

Beyond the BCL-2 family, senescent cells rely on multiple anti-apoptotic and proteostasis pathways for survival. N-myristoyltransferase inhibitors induce senescent-cell apoptosis by disrupting COPI vesicle formation and N-myristoylation, and improve disease-related outcomes in cancer and fibrosis mouse models (McHugh et al., 2023). This mechanism suggests potential relevance to fibrotic or matrix-remodeling-dominant skin lesions, although direct validation in skin disease is still lacking. The FOXO4-DRI peptide promotes senescent-cell apoptosis by releasing p53 from FOXO4 sequestration; in keloid organoids and fibroblast models, it clears senescent fibroblasts and reduces fibrotic signaling (Baar et al., 2017; Kong et al., 2025). The HSP90 inhibitor 17-DMAG (alvespimycin) was identified as a senolytic in Ercc1-deficient progeroid cells and progeroid mice, where it destabilizes AKT and related survival signals and improves healthspan-associated phenotypes (Fuhrmann-Stroissnigg et al., 2017). These strategies should therefore be viewed as senolytic candidates that exploit anti-apoptotic or proteostasis dependencies, rather than as conventional proliferation-pathway inhibitors. Their cutaneous translation should focus on models of scar fibrosis, chronic wounds, and post-photodamage matrix remodeling. These strategies should be viewed as senolytic candidates that exploit anti-apoptotic or proteostasis dependencies, rather than conventional proliferation-pathway inhibitors; their cutaneous translation should focus on models of scar fibrosis, chronic wounds, and post-photodamage matrix remodeling.

Plant-derived senolytic candidates remain mainly at the stage of mechanistic discovery, and their positioning in skin disease requires caution. Gingerenone A downregulates BCL-XL, activates caspase-3, and suppresses SASP secretion in senescent cells (Moaddel et al., 2022), suggesting combined pro-apoptotic and antisecretory potential. Cycloastragenol induces senescent-cell apoptosis through BCL-2 and PI3K/AKT/mTOR regulation in a non-cutaneous traumatic brain injury aging mouse model (Zhang et al., 2023), whereas indole-3-carbinol induces apoptosis in senescent fibroblasts with changes in BAX/BCL-2, TP53, and SIRT1 (Sax et al., 2024). These compounds may therefore be prioritized as candidates for skin aging, photodamage, or fibrotic disease, but their anti-inflammatory efficacy in psoriasis, AD, or chronic wounds cannot yet be inferred.

### 5.2 Senomorphics: modulating SASP

Senomorphics are intended to reshape the secretory phenotype of senescent cells rather than directly eliminate the entire population of stressed cells. For skin, a tissue continuously exposed to barrier injury, microbial stimuli, and mechanical stress, this approach is attractive because it may reduce chronic inflammation, proteolysis, and paracrine senescence while preserving the beneficial effects of transient senescence during wound repair. A reduction in SASP-associated mediators is not sufficient to define a senomorphic effect unless the cellular source and senescent state are demonstrated.

cGAS-STING-directed candidates are relevant because DNA damage and mitochondrial stress can drive the transition from a senescent state to an inflammatory secretory program through cGAS-STING. This pathway is therefore particularly relevant to photodamage, radiation injury, and chronic inflammation-associated skin aging. Ginkgetin directly binds STING and reduces SASP secretion and cell-cycle inhibitor expression in experimental aging models (Liu et al., 2025), providing preliminary support for its candidacy as a senomorphic. If related compounds improve psoriasis-like inflammation or UV injury without p16, p21, SA-β-gal, or cell-type-resolved SASP-lineage data, their disease relevance should still be classified as anti-inflammatory or photoprotective rather than senomorphic.

There is important overlap between general inflammatory-pathway inhibitors and senomorphics. NF-kappaB and JAK-STAT blockade can reduce multiple SASP-associated cytokines and may therefore have added value in lesions enriched for senescent cells. However, without evidence of cellular source, established immunomodulatory drugs should still be regarded as anti-inflammatory therapies rather than being directly equated with senescence-targeted drugs. Future studies combining treatment with spatial localization or single-cell-resolution assays will help determine whether standard anti-inflammatory therapies also weaken the secretory function of senescent cells.

mTOR regulators have more developed evidence in skin. Rapamycin currently has relatively clear human-skin senomorphic evidence: topical treatment reduces epidermal p16^INK4a^ expression and increases collagen VII content in aged skin (Chung et al., 2019). In skin fibroblast models, rapamycin also reduces SA-β-gal activity and regulates p53, MMP-1, and type I collagen alpha 1 expression (Tang et al., 2024); UVB photoaging models further suggest that it reduces fibroblast injury by lowering ROS (Qin et al., 2018). These findings mainly support use in photoaging and age-related skin degeneration and are not yet sufficient to infer efficacy in inflammatory skin disease.

p38 MAPK pathway modulators have clearer therapeutic relevance in aged skin immunity and radiation injury. Vitamin D3 partially inhibits p38 MAPK and reduces inflammatory mediators secreted by senescent fibroblasts; in human skin studies, vitamin D3 supplementation is also associated with improved VZV-specific cutaneous immunity in older individuals (Sayegh et al., 2024). Depending on context, vitamin D3 should be classified as a senomorphic candidate or a geroprotective agent rather than a broad senomorphic. Zileuton, a 5-lipoxygenase inhibitor, reduces SASP and cellular senescence-related markers and promotes ulcer improvement in rodent models of radiation-induced cutaneous ulcers (Park et al., 2022). These studies mainly support the potential application of p38/SASP modulation in age-related cutaneous immune decline and radiation-induced skin injury.

Plant-derived and metabolic senomorphics provide another class of candidate strategies for long-term skin management, but they should be classified according to whether they modify already senescent cells or prevent senescence. Rutin downregulates representative SASP factors in senescent cells, mainly supporting broad senomorphic potential (Liu et al., 2024). Liuweirunfu oil has been linked to BCAA-driven inflammatory senescence in AD relapse studies and includes p21 and SASP factor readouts, suggesting that metabolic reprogramming may influence inflammatory maintenance in recurrent AD (Wang Y. et al., 2025). It should be viewed as a candidate senomorphic in AD relapse rather than a general senomorphic. The shared value of these candidates lies in connecting environmental exposure, metabolic state, and the senescence secretory program, but their effects still need to be distinguished from simple antioxidant, barrier-repair, or conventional anti-inflammatory activity.

### 5.3 Senopreventive/geroprotective strategies

Some compounds prevent the establishment of senescence under stress rather than modifying already senescent cells. Evidence for metformin in skin remains preliminary, but studies in UVA-induced photoaging models have included senescence endpoints and show that metformin attenuates fibroblast senescence through regulation of mitophagy and PI3K/AKT/mTOR signaling (Chen et al., 2022). Hesperidin protects PM2.5-exposed HaCaT keratinocytes from mitochondrial damage, cell-cycle arrest, and cellular senescence, making it most appropriate for discussion in pollution-related skin stress (Herath et al., 2022). Magnolin attenuates CXCL10-driven and UV-related skin-aging phenotypes and affects p16, p21, β-galactosidase activity, and MMP expression, mainly supporting relevance to photoaging or inflammatory skin aging (Wang C. et al., 2025). Ganoderma lucidum extract modulates antioxidant-defense, cytoprotective, and p16-related transcriptional signals in human dermal fibroblasts (Kühnel et al., 2025), a profile more consistent with candidate protection against skin aging or photodamage. These effects are more accurately described as senopreventive or geroprotective than senomorphic. Antioxidants, vitamin D, and plant polyphenols that merely prevent stress-induced p16/SA-β-gal appearance should not be directly termed senomorphic; they are more likely senopreventive or geroprotective.

### 5.4 Lesion-directed delivery and emerging senescence-targeted strategies

The skin is one of the most suitable organs for developing lesion-directed senotherapeutics. Skin lesions are visible and can be sampled repeatedly, and topical treatment, intradermal injection, microneedles, and responsive nanocarriers can all limit systemic exposure to some extent. Because systemic toxicity and tissue selectivity remain major translational barriers for many senolytics, local delivery may not only improve drug access to lesions but also move senescence-targeted therapy from a systemic anti-aging concept toward precision treatment of defined cutaneous lesions.

Current representative delivery studies have focused mainly on diabetic wounds and psoriasis-like inflammation. The PARP1@PLS-PT100 system uses dipeptidyl peptidase 4 receptor recognition to target senescent fibroblasts and accelerates healing of diabetic ulcers in rats (Zhao et al., 2022). The F@GP system encapsulates Fe3O4 in galactose-modified poly (lactic-co-glycolic acid) and selectively induces ferroptosis of senescent fibroblasts through the Fenton reaction, thereby improving healing in diabetic wound models (Wei et al., 2023). In a psoriasis model, activatable photodynamic therapy combined with the PDE4 inhibitor crisaborole was designed to achieve both senolysis and immunomodulation (Yu Y. et al., 2025). These strategies indicate that senescence-targeted therapy can be combined with lesion-selective release, local immunomodulation, and conventional anti-inflammatory drugs to create therapeutic models more closely aligned with dermatological disease settings.

Beyond drug delivery, mobilizing local immune clearance is an emerging direction. Low-intensity pulsed ultrasound selectively stimulates senescent cells to produce SASP factors involved in immune recruitment, suggesting that it may act by enhancing immune recognition rather than by directly killing senescent cells (Gwak et al., 2025). If validated in skin-disease models, this approach would expand senescence-targeted therapy beyond killing senescent cells or suppressing SASP to include restoration of local immune surveillance and lesion remodeling. Artificial mitochondrial transfer, exosomes, broad CRISPR screens, and conventional light- or energy-based devices may remain exploratory directions, but they should not be included as current senotherapeutic evidence until they are shown to reduce senescent-cell burden or alter SASP sources in cutaneous lesions.

## 6 Translational pathways and future directions

Translating cellular senescence into a therapeutic target requires more than documenting marker-positive cells. Studies must establish whether a defined lesion-resident population sustains disease and whether its local modulation improves pathology without compromising repair or immune defense.

### 6.1 Defining senescence-driven lesions

A central unresolved issue is how to distinguish a driving role for senescent cells from their accompanying accumulation in disease. Inducible genetic clearance models such as p16-3MR and INK-ATTAC can be used to test whether p16^INK4a^-positive cells participate in disease initiation, persistence, or repair failure. Pharmacological senolytic studies should be combined with single-cell or spatial omics to track dynamic changes in senescent-cell populations, SASP sources, and the state of neighboring immune cells before and after treatment. Comparing senolytics, senomorphics, and standard anti-inflammatory drugs within the same disease model would help define the boundary between the specific benefit of senescence-targeted intervention and general immunosuppression.

Human studies should therefore use a tiered, multiparameter strategy rather than treat individual markers as interchangeable. At minimum, translational assessment should combine evidence of cell-cycle arrest (p16^INK4a^ or p21^CIP1^ together with reduced proliferative activity, such as low Ki-67) with at least two auxiliary hallmarks, with co-detection in the same cells whenever technically feasible (Ogrodnik et al., 2024; Suryadevara et al., 2024). Auxiliary structural and lysosomal evidence may include loss of LMNB1 and lipofuscin-based staining in fixed tissue; cell or nuclear enlargement is less robust in skin sections, whereas SA-β-gal is most reliable in fresh or frozen samples (Ogrodnik et al., 2024; Suryadevara et al., 2024). Transcriptional identity should be resolved by combining lineage markers with cell-type-specific senescence and SASP programs. Skin-specific signatures such as SenSkin can support this analysis (Wyles et al., 2025). Single-cell and spatial transcriptomic analyses can localize candidate senescent populations to epidermal or dermal compartments (Yu G. T. et al., 2025), while spatial transcriptomic or proteomic approaches can further assess their proximity to immune cells and other potential responders (Suryadevara et al., 2024). However, because these skin-specific resources have been evaluated mainly in aging and photoaging rather than inflammatory dermatoses, orthogonal protein or histological confirmation remains necessary.

### 6.2 Defining treatment windows

Senescence-targeted treatment should be defined by cell type, disease phase, duration, and anatomical compartment. In acute wounds, transiently senescent fibroblasts and endothelial cells support granulation tissue formation through PDGF-AA; indiscriminate clearance during early repair may therefore disrupt healing (Demaria et al., 2014). In contrast, human chronic wounds show associations between senescence markers and delayed healing (Yu et al., 2024), while diabetic-wound models provide direct preclinical support for targeting senescent fibroblasts and for broader senescent-cell clearance (Samarawickrama et al., 2024; Zhao et al., 2022). Intervening at the appropriate disease stage is critical to preserve the beneficial effects of transient senescence while limiting persistent senescent-cell accumulation. However, the optimal timing and duration of treatment have not been systematically established, and future studies should compare early versus delayed intervention and single-dose versus intermittent regimens. Although local and cell-selective senescence-targeted approaches have shown benefits in experimental wound models, their effects across distinct anatomical compartments have not been systematically evaluated. Future studies should determine whether persistent senescent cells in the wound bed or pathological dermis can be targeted while sparing reparative cells at the migrating epidermal edge, functional vasculature. For psoriasis, AD, and other inflammatory dermatoses, the optimal target cell, disease phase, treatment duration, and anatomical compartment remain undefined.

### 6.3 Improving disease models and intervention safety

Preclinical models need to more closely approximate human pathology. Mouse skin differs from human skin in immune composition, tissue architecture, and inflammatory chronicity (Gerber et al., 2014; Pasparakis et al., 2014), and senotherapeutics often act only on senescent states in specific cell types (Pils et al., 2021). Candidate drugs should therefore be prioritized for validation in models matched to the target disease, such as diabetic or aged wounds, photodamaged skin, or inflammatory lesions with clear senescent-cell enrichment. The safety window will also determine translational feasibility: rapamycin can suppress general immune function (Dumont and Su, 1996), dasatinib can affect hematopoiesis (Feld et al., 2022), and BCL-2/BCL-XL inhibitors carry hematological toxicity risks.

### 6.4 Integrating host and environmental variables

Cutaneous senescence is shaped by systemic and environmental factors. The gut-skin axis, microbial metabolites, psychological stress, hyperglycemia, dietary patterns, and UV exposure can all influence redox state, matrix remodeling, and immune activation (Boyajian et al., 2021; Cao et al., 2025; Lorenzo et al., 2023). AGEs accumulate in long-lived dermal proteins and can be estimated using methods such as skin autofluorescence. High-glucose, high-fructose, or AGE-rich environments can enhance oxidative and inflammatory signaling and may promote senescence-like stress in diabetic or chronically inflamed skin (Papagrigoraki et al., 2017b; Rella et al., 2025; Wang et al., 2024). High-sugar, high-salt, or high-fat diets can further aggravate skin aging through oxidative injury and NLRP3 inflammasome activation (Herbert et al., 2018; Kreouzi et al., 2025; Liang et al., 2022; Peng et al., 2024). These variables may influence patient stratification and efficacy analyses in future senescence-targeted trials.

## 7 Conclusion

Overall, cellular senescence provides a mechanistic framework for understanding skin inflammation and tissue injury across stress sensing, SASP output, immune clearance, and tissue-compartment remodeling. Its central relevance lies in explaining why some lesions may shift from transient repair responses to sustained chronic inflammation. Current evidence is strongest in chronic diabetic ulcers and aged wounds, where senescent-cell accumulation, SASP expression, and clearance interventions have been linked to healing outcomes. In psoriasis, AD, photoaging, and other lesions, senescence-associated signals suggest possible involvement in disease maintenance, but the strength of evidence varies and remains insufficient in common inflammatory skin diseases to establish a causal role in disease development, severity, or therapeutic response. Future clinical translation should proceed in two main directions: first, using senescence markers and spatial maps to identify lesion subtypes that are genuinely driven or maintained by senescent cells; and second, developing local senolytic or senomorphic strategies for these subtypes. If future studies demonstrate that altering senescent cells or their secretory programs within lesions improves inflammation and promotes matrix repair, senescence-targeted therapy may become an important adjunct to existing immunomodulatory approaches.

## Glossary

4-HNE: 4-hydroxynonenal
AD: atopic dermatitis
AGE: advanced glycation end product
AKT: protein kinase B
ATM: ataxia-telangiectasia mutated
ATR: ataxia telangiectasia and Rad3-related
BCAA: branched-chain amino acid
BCAT1: branched-chain amino-acid transaminase 1
BCL-2: B-cell lymphoma 2
CCL20: C-C motif chemokine ligand 20
CCN2: cellular communication network factor 2
cGAS: cyclic GMP-AMP synthase
CHK: checkpoint kinase
CLE: Cutaneous lupus erythematosus
CXCL: C-X-C motif chemokine ligand
CXCR2: C-X-C motif chemokine receptor 2
DFU: diabetic foot ulcer
DPP4: dipeptidyl peptidase 4
ECM: extracellular matrix
EGFR: epidermal growth factor receptor
EndMT: endothelial-to-mesenchymal transition
FOXO4-DRI: FOXO4-D-retro-inverso peptide
HLA-E: human leukocyte antigen E
HLA-I: human leukocyte antigen class I
HP1: heterochromatin protein 1
IFN: interferon
IGFBP2: insulin-like growth factor binding protein 2
IGFBP2: insulin-like growth factor binding protein 2
IMQ: imiquimod
IRF3: interferon regulatory factor 3
JAK-STAT: janus kinase-signal transducer and activator of transcription
MAPK: mitogen-activated protein kinase
MDA: malondialdehyde
MICA/MICB: MHC class I chain-related proteins A and B
miMOMP: minority mitochondrial outer membrane permeabilization
MITF: microphthalmia-associated transcription factor
MMPs: matrix metalloproteinases
mTOR: mechanistic target of rapamycin
NB-UVB: narrowband ultraviolet B
NCOA4: nuclear receptor coactivator 4
NF-κB: nuclear factor kappa-B
NK: natural killer
NKG2A: natural killer group 2 member A
NKG2D: natural killer group 2 member D
NLRP3: NOD-like receptor family pyrin domain containing 3
PAH: polycyclic aromatic hydrocarbon
p21: p21^CIP1^
PDE4: phosphodiesterase 4
PD-1: programmed cell death protein 1
PD-L1: programmed death-ligand 1
PDE4: phosphodiesterase 4
PDGF-AA: platelet-derived growth factor AA
PDK4: pyruvate dehydrogenase kinase 4
PI3K: phosphoinositide 3-kinase
PM2.5: particulate matter ≤2.5 μm
pRB: retinoblastoma protein
RAGE: receptor for advanced glycation end products
ROS: reactive oxygen species
RPA: replication protein A
SA-β-gal: senescence-associated beta-galactosidase
SASP: senescence-associated secretory phenotype
SIRT: sirtuin
SSc: systemic sclerosis
STAT: signal transducer and activator of transcription
TET2: ten-eleven translocation 2
TL: telomere length
uPAR: urokinase plasminogen activator receptor
UV: Ultraviolet
VZV: varicella-zoster virus
YAP: Yes-associated protein
17-DMAG: alvespimycin
